# Immediate Breast Reconstruction in Skin-Reducing Mastectomy with Prepectoral Polyuretane (Pu) Implant Covered with an Autologous Dermo-Adipose Flap

**DOI:** 10.1007/s00266-022-03240-8

**Published:** 2023-01-11

**Authors:** Fedele Lembo, Liberato Roberto Cecchino, Domenico Parisi, Aurelio Portincasa

**Affiliations:** Department of Plastic and Reconstructive Surgery, Foggia Medical School, University of Foggia, via Pinto, 71121 Foggia, Italy

**Keywords:** Prepectoral, Polyurethane, Reconstruction, Skin-reducing mastectomy, BREAST-Q

## Abstract

**Background:**

The aim of this study was to present our new technique of immediate breast reconstruction with prepectoral Polyuretane (PU) Implants, covered with an autologous dermo-adipose flap, in skin-reducing mastectomy both for risk-reducing (prophylactic mastectomy) and therapeutic cases.

**Methods:**

We performed a single-center, retrospective review of 21 patients (mean age 47 years), undergone skin-reducing mastectomy and immediate breast reconstruction with prepectoral Polyuretane (PU) Implants, covered with an autologous dermo-adipose flap, un the period January 2018–June 2021. All procedures were performed by the same surgeon.

**Results:**

A total of 36 skin-reducing mastectomies (6 curative, 15 prophylactic) with one-stage prepectoral PU foam-coated implant and autologous dermo-adipose flap reconstruction were performed. All postoperative complications were collected. Quality of life (QoL) and satisfaction with reconstruction were evaluated through the BREAST-Q questionnaire, administered preoperatively and at 12 months after surgery. Independent Student’s *t* tests were used to compare means of continuous variables and Chi-square test was used for ordinal variables. A *p*-value ≤0.05 was considered statistically significant. Uni- and multiple linear regression analyses were used to confirm all results. Out of 21 patients, we observed one small wound dehiscence and one partial Nipple-Areola Complex (NAC) necrosis. All cases of minor complications were managed conservatively and did not affect the quality of the final outcome. No implant loss was observed. No significant severe capsular contracture (grade III or IV) was detected at follow-up. Overall satisfaction with breasts, psychosocial, physical and sexual well-being all significantly increased, analyzing BRAST-Q data. Statistical analysis showed a significant influence of diabetes on the risk of complications.

**Conclusions:**

Our experience suggests that the procedure described is effective, feasible and cost-effective. It is easier to perform compared to similar and more demanding procedure, reduces operative time, and minimizes complications related to manipulation of the pectoralis major muscle, while also contributing to the containment of costs.

Moreover, it appears to be oncologically safe, provides good esthetic results with low postoperative complication rate and leads to high level of patients’ satisfaction.

**Level of Evidence IV:**

This journal requires that authors assign a level of evidence to each article. For a full description of these Evidence-Based Medicine ratings, please refer to the Table of Contents or the online Instructions to Authors www.springer.com/00266.

## Introduction

Immediate implant-based mammary reconstruction is one of the most common approach for surgical treatment of patients undergoing skin-reducing mastectomy (SRM) both for breast cancer treatment and for prophylactic aims, in women at oncologic high risk to develop breast cancer[1–4].

Direct prosthetic breast reconstruction, in selected cases, is fundamental to restore immediately women body image and self-esteem, with very high positive psychological effects to the patients.

In the last decade, the plastic surgical approach to these patients has radically changed; in fact, many Plastic Surgeons switched from subpectoral implant placement to prepectoral positioning of the prosthesis [5]. Although subpectoral implant placement might offer better prosthetic protection and lower risk of capsular contracture (CC), on the other hand the prepectoral approach presents many advantages: it is a less invasive and a muscle sparing procedure, guarantees less postoperative pain, eliminates breast animation deformity and potential impairment to shoulder motion, allows less operative time and hospital stay [6]. Even if various Authors argued on the need of using biological Acellular Dermal Matrix (ADM) or synthetic meshes in order to cover the prepectoral implant, nevertheless an increased risk of seroma, extrusion, infection and much higher costs have been reported in the literature. [5, 7]

In order to reduce these disadvantages, some works have recently shown that polyurethane foam-covered implants (Microthane^®^, Polytech, Dieburg, Germany) can be safely implanted in a prepectoral plane without using neither ADM nor synthetic meshes. [8–14]

The combination of prepectoral positioning of polyurethane implants can guarantee an optimal, reliable and cost-effective reconstruction in skin-reducing mastectomy (SRM).

The aim of this study is to present our new technique of immediate breast reconstruction with prepectoral Polyuretane (PU) Implants, covered with an inferiorly based autologous dermo-adipose flap, to protect the implant and lower overall the complication rate, in skin-reducing mastectomy both for risk-reducing (prophylactic mastectomy) and therapeutic cases.

## Materials and Methods

### Data Collection

Patients’ data were recorded including age, BMI, smoking habitus, comorbidities, ASA score, prior surgery, radiotherapy or chemotherapy, indication to surgery (therapeutic or prophylactic), time of procedure, weight of breast removed, implant size, axilla management (Sentinel Linfo Node Dissection (SLND) or Axilla Linfo Node Dissection (ALND)), and adjuvant treatment. (View Table1)

**Table 1:** Patients’ data

Characteristics	Values
No. of patients	21
No. of breasts	36
Average age, years [range]	47 [30–66]
Average BMI (kg/m^2^) [range]	26.94 [24.3–35.6]
Diabetes	3
Smoking	5
Hypertension	5
Anemia	1
Dyslipidemia	6
ASA 1	10
ASA 2	11
*Indication for surgery*	
Therapeutic	6
Prophylactic	15
*Axylla management*	
SLND	6
ALND	0
Neoadjuvant treatment	1
Adjuvant treatment	1
Average nipple-to-sternal notch distance (SD) [range]	32.2 (1.9) [26.9–35.3]
Mean hospital stay (SD) [range]	2.5 (0.8) [2–4]
Average follow-up, months (SD) [range]	19.3 (2.8) [12–53]
Average time of surgical procedure (min)	190 [150–230]
Mastectomy specimen weight (g)	385 [320–690]
Average implant volume (cc) [range]	445[380–550]
NAC graft	15
NAC flap	6

In all patients micro-polyurethane foam-coated anatomical breast implants (Microtane^®^ Polytech, Germany) were used.

All patients were followed with periodic controls at 1, 3, 6, 12, 18 and 24 months.

All postoperative complications such as: hematoma, seroma, wound dehiscence, partial/total nipple-areola complex (NAC) sufferance/necrosis, implant exposure/loss, severe capsular contracture, rippling and red breast syndrome were recorded and analyzed. (View Table2)

**Table 2:** Surgical complications

Type of complication *n*, % (95%CIs) [3]	Patient’s level [1] *N* = 21	Breast’ level [2] *N* = 36
Wound dehiscence	1 4.8% (0.1–23.8)	1 2.8% (0.0–14.5)
Partial NAC necrosis	1 4.8% (0.1–23.8)	1 2.8% (0.0–14.5)
Complete NAC necrosis	0 0.0% (0.0–16.1)	0 0.0% (0.0–9.7)
Hematoma	0 0.0% (0.0–16.1)	0 0.0% (0.0–9.7)
Seroma	0 0.0% (0.0–16.1)	0 0.0% (0.0–9.7)
Rippling	0 0.0% (0.0–16.1)	0 0.0% (0.0–9.7)
Severe capsular contracture	0 0.0% (0.0–16.1)	0 0.0% (0.0–9.7)
Implant loss	0 0.0% (0.0–16.1)	0 0.0% (0.0–9.7)
Red breast syndrome	0 0.0% (0.0–16.1)	0 0.0% (0.0–9.7)

The number of patients with at least of one complication in one of the breasts is shown. Percentages are calculated on the number of patients (*N*). The number of breasts with at least of one complication is shown. Percentages are calculated on the number of breasts (*N*). 95% Confidence intervals are calculated with the Clopper–Pearson’s exact method.

Quality of life (QoL) and patient satisfaction after reconstruction were evaluated through the BREAST-Q questionnaire, administered preoperatively and at 12 months after surgery.

Statistical analyses were conducted using the Statistical Package for Social Sciences (SPSS) Software for Windows version 23. Independent Student’s *t* tests were used to compare means of continuous variables and Chi-square test was employed for ordinal variables. A *p*-value ≤0.05 was considered statistically significant. Uni- and multiple linear regression analyses were used in order to confirm all results.

### Patient Selection

In the period January 2018–June 2021, 21 consecutive patients underwent prophylactic or therapeutic skin-reducing mastectomy and immediate Direct To Implant (DTI) breast reconstruction with prepectoral positioning of polyurethane foam-coated implants covered with an inferiorly based autologous dermo-adipose flap. Inclusion criteria were: women aged ≥18 years, mono- or bilateral prophylactic (for breast cancer-related gene mutation) or therapeutic (for breast cancer) SRM. Exclusion criteria were: harder smoker and autoimmune diseases or immunosuppressive therapy. Obesity, diabetes and radiotherapy were not considered as contraindications. The study was conducted according to the Declaration of Helsinki and all patients signed an informed consent to the procedure. All procedures were conducted by the same surgeon.

### Surgical Technique

Our surgical technique consisted of a combination of a classic skin-reducing mastectomy (involving the reduction of the skin envelope and the de-epithelization of a lower dermo-adipose flap) with implant of a polyurethane foam-coated prosthesis in the prepectoral plane, without detachment of the large pectoral muscle and without implant covering with any ADM or synthetic meshes.

Both the mastectomy and the reconstructive procedures were performed under general anesthesia by the same surgeon.

In all patients we performed a Wise nipple sparing skin-reducing mastectomy with de-epithelization of an inferior dermo-adipose flap (approximately 10 cm in height and 15 cm in length), related both to the footprint and to the width of the breast. The nipple-areola complex (NAC) was transposed on a superiorly based pedicle (if notch-NAC distance range was ≤29 cm), while it was harvested as a full-thickness skin graft when the distance was >29 cm. In all cases, intraoperative fresh-frozen examination of the retroareolar tissue was performed before completing the reconstruction.

**Fig. 1:**
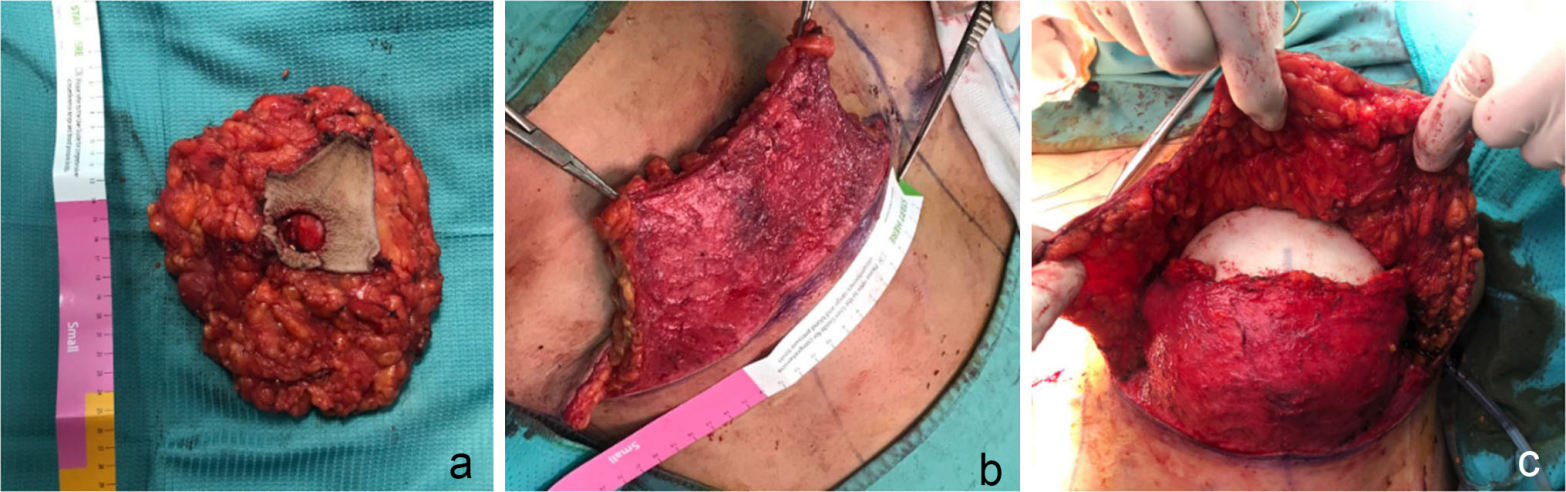
**a** Anatomical specimen of mastectomy; **b**. inferior dermo-adipose sling; **c**, PU implant positioned under the dermo-adipose inferior sling. Note the thickness and the vitality of mastectomy flaps

In all mastectomies, we proceeded prior with a dermal infiltration of 50 cc of a solution containing 1mg/ml epinephrine in 1L of NaCl, followed by scissor dissection to obtain the inferior dermo-dipose flap. All mastectomies were performed using an ultrasonic scalpel (Focus Ultracision Harmonic Scalpel^®^, ETHICON, Johnson-Johnson, Belgium). SLNDs were performed through a separate axillary incision or through the mastectomy incision, depending on SLNDs location, breast volume and width. NAC and mastectomy flap perfusion were clinically evaluated based on color, capillary refill, dermal bleeding. The prepectoral pocket was systematically inspected to be sure about the total removal of breast tissue. In all cases we proceeded with the direct implant of a polyurethane foam-coated prosthesis in the prepectoral plane, covering it with the inferiorly based dermo-adipose flap. (See Fig. 1) The implant selection was based on preoperative measurements, skin quality, and contro-lateral breast parameters such as: size, volume, projection and footprint; before the definitive implant positioning, we used sizers to confirm the choose with the patient set in upright 60° sitting position. The definitive implant, positioned in the prepectoral plane, was covered by the adipo-dermal flap preserved previously, without any tension; this maneuver leads to several advantages: strengthen of the lower quadrants in a fashion of “autologous bra”, protect the implant with a double layer covering it in the junction point of the “inverted T” scar, allows a safe closure with no tension on the pattern skin flaps not to impair their vascularization, lowers the risk of dehiscence, ensure better healing, thus allowing higher quality of the final scars. One 10 F drainage (Blake^®^, Ethicon, Johnson-Johnson, USA) was routinely placed in the prepectoral pocket, prior to implant insertion, keeping it between the inferior border of the prosthesis and the underlying anterior fascia of the pectoralis major muscle. The pattern skin flaps were draped and sutured centrally and inferiorly in layers with Monocryl 3/0, in total absence of tension. Preoperative short-term antibiotic prophylaxis with Cefazolin 2 g was used in all patients. The drains were removed when their daily output was less than 40 ml in 2 days. After discharge, the follow-up was planned and all patients were instructed to wear a specific postoperative bra day and night for at least 40 days.

## Results

Twenty-one patients were operated with a mean age of 47 years (from 30 to 66 years; SD 12.37) and a mean BMI of 26.94 kg/m^2^ (24.3−35.6; DS 4.12). Fifteen patients (*n*=15/21; 66.7%) underwent bilateral reconstruction. In six patients (28.6%) the procedure was curative, whereas in the remaining 15 (71.4%) the procedures were prophylactic. Seven patients presented other comorbidities: three cases of diabetes (*n*=3/21; 14%), five cases of arterial hypertension (*n* = 5/21; 23.8%), one case of anemia (*n*=1/21; 4%), and six cases of dyslipidemia (*n* = 6/21; 28.6%).

Four patients were previous smokers (smoking was stopped more than 6 months before surgery; *n* = 4/21; 19.0%), while five were active but light smokers (less than 5 cigarettes/day) (*n* = 5/21; 23.8%).

In prophylactic mastectomies we observed: ten patients (*n* = 10/15; 66.67 %) presented with a BRCA-1 gene mutation, three with a BRCA-2 gene mutation (*n* = 3/15; 20%), 1 with a PTEN gene mutation (*n* = 1/15; 6.67%), and one presented with a TP53 genetic mutation (*n* = 1/15; 6.67%).

In six cases of curative SRM we removed five ductal carcinomas (four cases T1 and one case T2 stage) and one lobular carcinoma (T1 stage).

Ten patients were ASA I, 11 patients were ASA II.

One patient benefited from neoadjuvant treatment (*n* = 1/21; 4.7%) with chemotherapy, and in two cases (*n* = 2/21; 9.5%) we reported a story of previous surgery and local radiotherapy (QUART).

In total, 36 skin-reducing mastectomies were performed with a mean specimen weight of 385 g (320–690; SD: 54.3). In all cases anatomical-shaped silicone implants foam PU coated, covered with the inferiorly based dermo-adipose flap were employed, with an average size of 445 cc (range, 380–550 cc). The operative time for mastectomy and DTI reconstruction was on average 190 minutes (range, 150–230 minutes). The NAC was transposed based on a superiorly based pedicle in six patients (if notch-NAC distance range was ≤29 cm), while it was harvested as a full-thickness skin graft in 15 cases (when nipple-areola distance was >29 cm).

Six SLNDs (*n* = 6/21; 28.6%) and no ALND were performed.

Measured cup sizes were distributed as follows: 15 D-cup (*n* = 15/21, 66.7%), and 6 E-cup (*n* = 6/21, 28.6%).

One patient (*n* = 1/21; 4.7%) received adjuvant radio treatment.

**Fig. 2:**
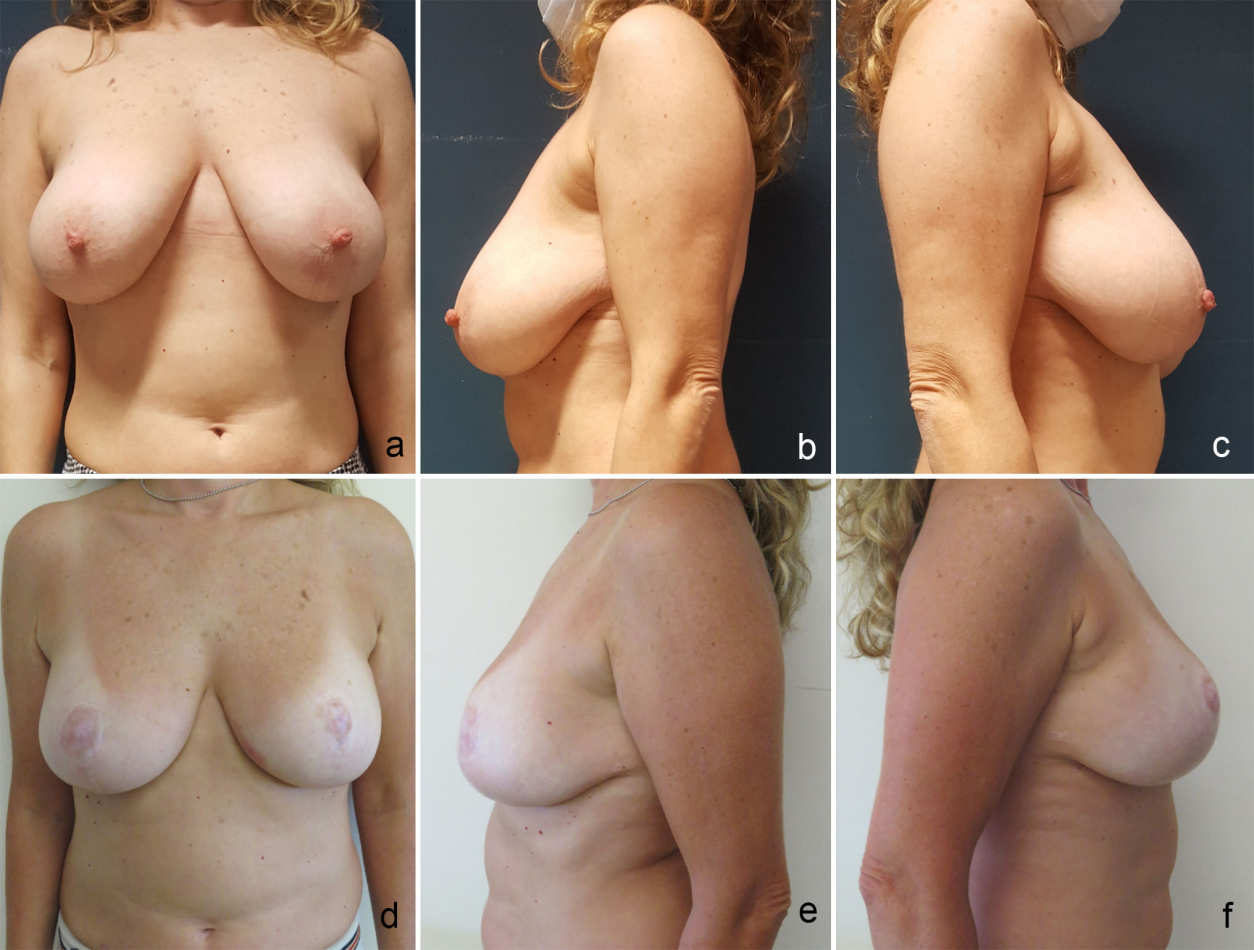
**a**, **b** and **c**. Preoperative frontal and lateral view. **d**, **e**, and **f**, Postoperative frontal and lateral view

**Fig. 3:**
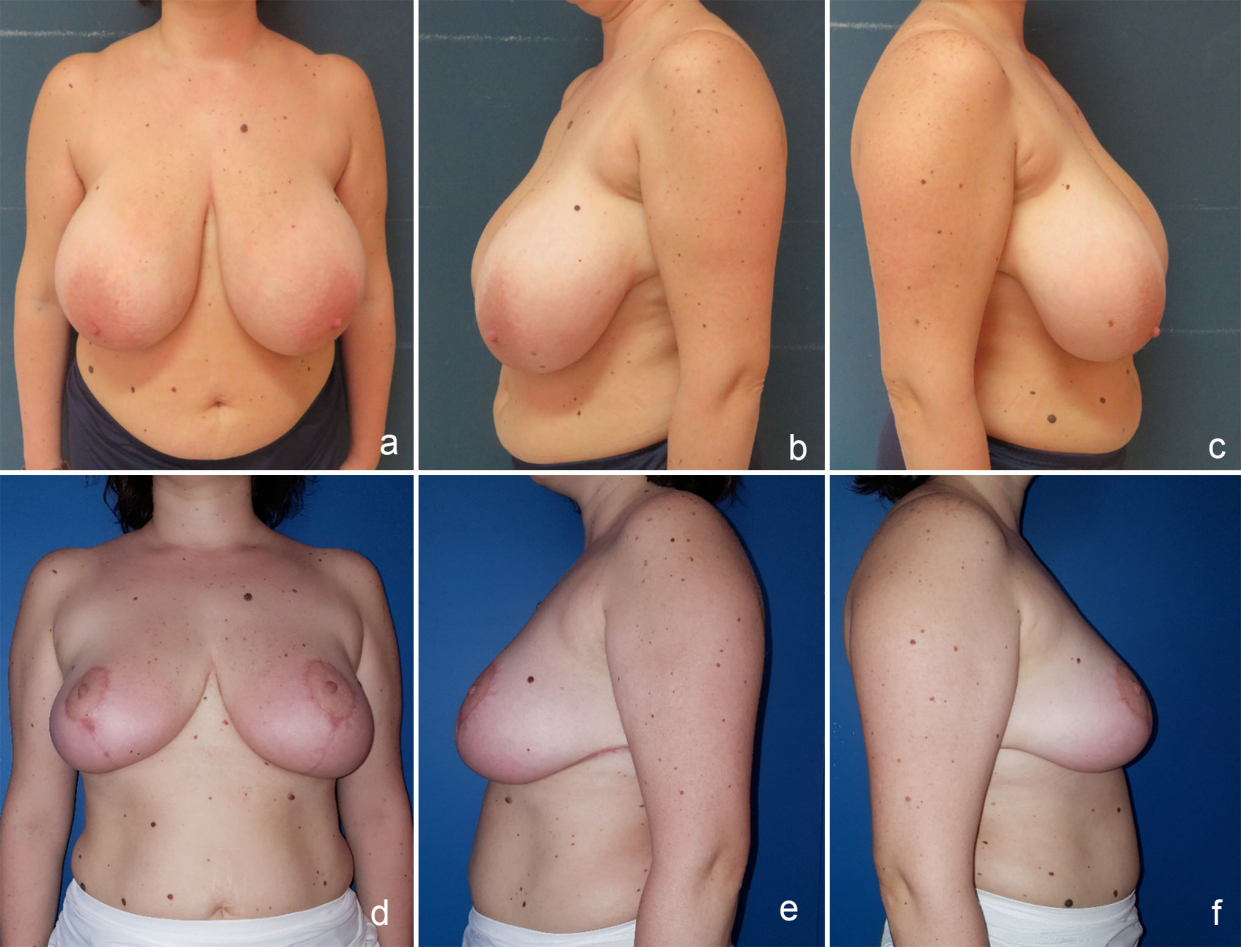
**a**, **b** and **c**. Preoperative frontal and lateral view. **d**, **e** and **f**, Postoperative frontal and lateral view

Pre- and postoperative photographs are shown in Figs. 2 and 3 .

The patients were discharged 2 days after surgery (mean hospital stay 2.5 days, range: 2–4).

Mean follow-up time was 19.3 months (range:12–53).

**Fig. 4:**
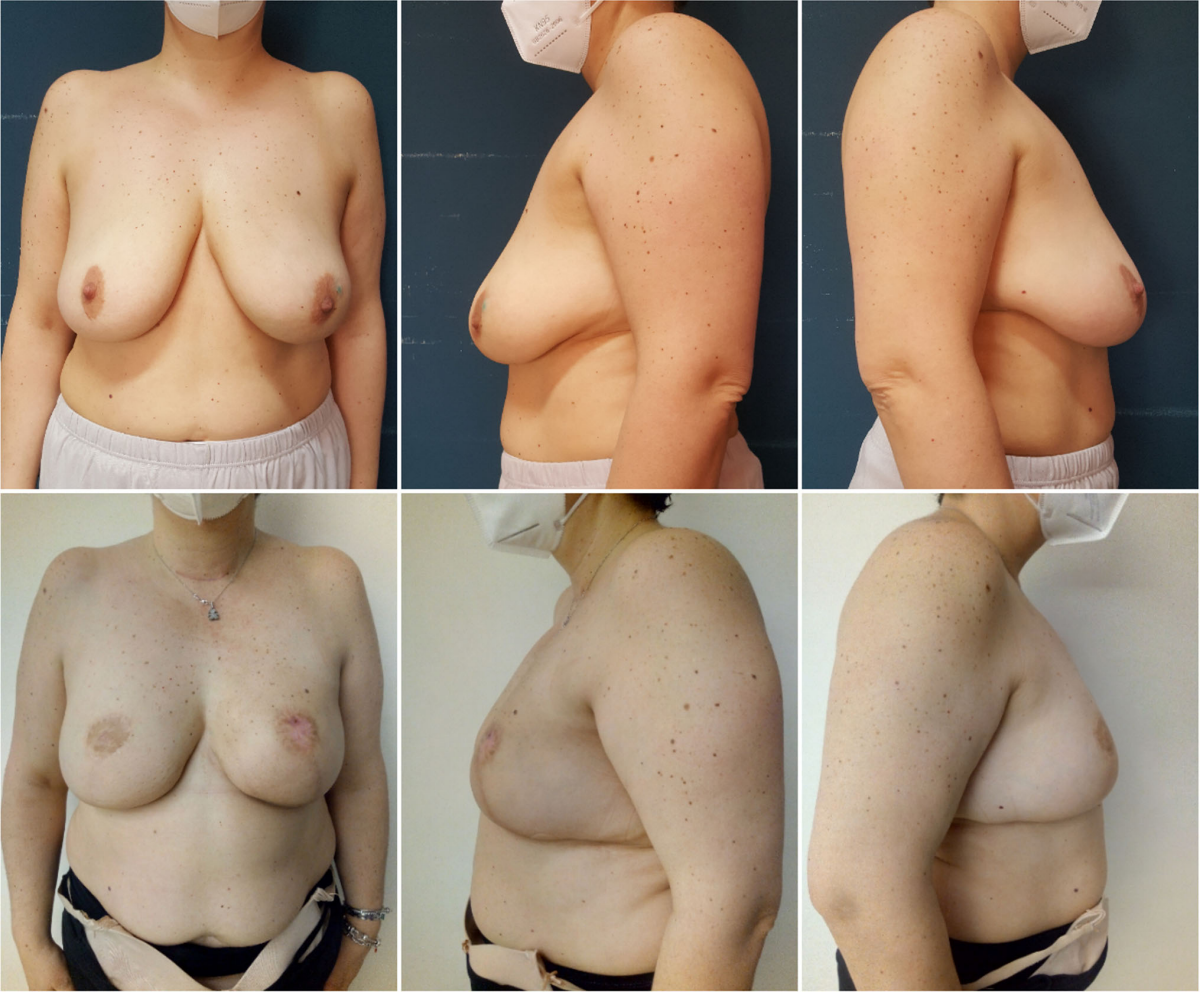
Postoperative frontal view. Note the partial necrosis of left NAC

Minor complications occurred in two breasts: one small wound dehiscence and one partial NAC necrosis (Fig. 4). All these cases of minor complications were managed conservatively, they did not determine a return to the operating room and did not compromise the final outcome both from the shape and esthetic point of view. No implant loss was observed. No significant severe capsular contracture (grade III or IV) was detected at follow-up.

BREAST-Q questionnaire results are reported in Table 3. Overall satisfaction with breasts, psychosocial, physical and sexual well-being all significantly increased after SMR and reconstruction (*p *< 0.05).

**Table 3:** BREAST-Q questionnaire

Domain	preop			postop1 year			*p* value
	*N*	Mean	SD	*N*	Mean	SD	
Breast Satisfaction	21	69.32	8.6	21	78.35	5.6	<0.05
Psychosocial well-being	21	71.43	6.3	21	89.22	9.7	<0.05
Physical well-being	21	67.74	3.5	21	77.21	3.4	<0.05
Sexual well-being	21	58.91	4.3	21	70.85	6.2	<0.05

No patients undergoing bilateral risk-reducing NSM in this series developed breast cancer during follow-up. Four patients underwent an additional lipofilling procedure 1 year after surgery, in order to improve the esthetic outcome.

Statistical analysis showed a significant influence of diabetes on the risk of complications, as shown in Table 4. In fact, using a univariate binary logistic regression diabetes seems to increase the risk of complication (*p*-value: 0.041). The influence of other variables (as previous radiotherapy, smoke, volume of breast, etc.) was not confirmed to be significant using binary logistic regression.

**Table 4:** Influence of variables on the risk of complications

Variable	Type of variable	Test used	Chi square/*t* test *p* value	Regression *p* value
ASA status	Ordinal	Chi Square	0.562	0.872
Diabetes	Dichotomous	Chi Square	0.024	0.041
HTA	Dichotomous	Chi Square	0.417	0.987
Active smoker	Dichotomous	Chi Square	0.855	0.652
Anemia	Dichotomous	Chi Square	n.r.	n.r.
Dyslipidemia	Dichotomous	Chi Square	0.746	0.988
Age	Continuous	Independent *T* Test	0.552	0.352
BMI	Continuous	Independent *T* Test	0.159	0.147
Neoadjuvant treatment	Dichotomous	Chi Square	nr	nr
Previous radiotherapy	Dichotomous	Chi Square	0.145	0.999
Adjuvant treatment	Dichotomous	Chi Square	nr	nr
SLND	Dichotomous	Chi Square	0.208	0.925
Breast weight	Continuous	Independent *T* Test	0.549	0.225
Therapeutic versus prophylactic	Dichotomous	Chi Square	0.809	0.687

## Discussion

In our study, skin-reducing mastectomy followed by DTI prepectoral reconstruction with PU foam-coated implants covered with the inferiorly based dermo-adipose flap was associated with low short- and long-term complications, high patients’ satisfaction and optimal long-term outcomes. This single stage reconstructive technique seems to be minimally invasive, with early discharge, rapid recovery and lower global costs.

The benefits of the prepectoral reconstruction have been widely discussed in the literature and consist of one-stage reconstruction, reduction of postoperative pain and discomfort, reduction in capsular contracture rates, better esthetic results without animation deformity.

However, high complication rates of seroma and expensive costs could hinder their use, if ADMs or meshes are used. The introduction of PU foam-covered implants in prepectoral reconstruction guarantees some advantages such as: prevention of implant displacement (the implant stays fixed to the chest wall without the need for mechanical support as meshes or ADMs); lower the rate of seroma and lower the costs (devices, days of hospitalization, time of surgery, etc.) rather than ADM prepectoral reconstruction. Several studies attest PU foam-covered implants advantages, reliability, safety and extremely low incidence of capsular contracture.

In fact, in 1991, Surgitek, a manufacturer of PU covered implants, voluntarily removed these implants from the US market for the alarming experimental data on cancers detected in animals overloaded with 2,4-toluenediamine (2,4-TDA), a breakdown product of PU [15, 16].

In 1995, the FDA affirmed the safety of PU implants based on the Bristol Mayers Squibb study that considered the previous data unreliable [17].

Also Hester et al [18] demonstrated the negligible risk of cancer (1,1/1 million) evaluating the serum and urine concentration of 2,4-TDA in women with PU implants.

Anyway, since PU foam-covered implant needs an immediate properly positioning because it stays fixed on the surrounding tissue as a “Velcro” effect, even in the long run, the surgeons should have a learning curve in order to avoid malpositioning of the prosthesis; on the other hand, in our technique, the prosthesis’ positioning is simple because the prepectoral pocket is wide and under direct view of the operator. Furthermore, the “adhesivity” of PU implants prevents the rotation and the friction between the surrounding tissue, with a lower risks of seroma and hematoma.

Moreover, the use the inferiorly based dermo-adipose flap covering the PU implant, stabilize it to the underlying superficial fascia of the pectoralis major muscle.

Another advantage of this technique is that no dead spaces or cavities are left at the end of the procedure, since both the dermo-adipose flap and the superficial flaps can be precisely tailored and closed over the PU implant as it is advisable.

These could be the reasons why, in our study during the follow-up, no major complications were encountered, no significant capsular contracture (grade III or IV), visibility of the superior pole of the implant and rippling have been observed in a mean follow-up of 24 months.

An adequate mastectomy flap that preserves both the dermal and the subcutaneous layer perfusion is the key to success of this surgery. In our clinical series tissue perfusion was assessed both clinically based on color, absence of dermal exposure and flap damage from diathermy; in doubt we preferred a delayed two steps reconstruction using a tissue expander and these patients were excluded from our study.

Careful patient selection is also crucial for a successful reconstruction lowering overall complications. Common risk factors are obesity, smoke, diabetes and large and ptotic breasts. However, some Authors reported better results of prepectoral reconstruction compared with subpectoral in high BMI women [19, 20]. In our work, also, breast volumes and BMI were not associated with an increasing of complications rate.

Radiotherapy in our series had no significant influence on complications. This is just a statistical statement. Most likely, one patient alone is not enough to draw a conclusion about the effect of radiotherapy on this type of implant reconstruction. A specific study is needed aimed at evaluating the clinical effects of radiotherapy (such as severe capsular contracture) on this new breast reconstructive technique, although further studies have shown that adjuvant radiotherapy appears to be well tolerated in immediate prepectoral breast reconstruction [21–26].

We have to underline two main limitations of this work. The first is related to the relatively short follow-up that might have prevented to note certain long-term complication, thus producing the so-called immortal time bias. In order to support this results, a longer follow-up are needed. The second limitation is linked to the fairly small sample size that might have affected the estimation of the complication rates. However, the 95% confidence intervals reported in Table 2 could help the reader to convey the grade of uncertainty associated to the sample size.

Our work represents the first report in the literature on patients undergone prepectoral immediate breast reconstruction with PU implants covered with an autologous inferiorly based dermo-adipose flap, following therapeutic or prophylactic skin-reducing mastectomy.

## Conclusion

Our study showed that prepectoral DTI reconstruction with PU foam implants, covered with an autologous inferiorly based dermo-adipose flap, without need of synthetic or biological meshes, in skin-reducing mastectomy is an effective, feasible and cost-effective procedure. It is easier to perform, reduces operative time, minimizes complications related to manipulation of the pectoralis major muscle, to the use of ADM or meshes, contributing to the containment of costs.

Moreover, it appears to be oncologically safe and provides good esthetic results with low postoperative complications rate and high level of patient satisfaction. Accurate patient selection, adequate surgical experience, and careful management of mastectomy flaps are fundamental to reduce the risk of postoperative complications. Further studies, with a larger population and longer follow-up are needed, in order to support our results.
